# Association of Neonatal Jaundice with Gut Dysbiosis Characterized by Decreased Bifidobacteriales

**DOI:** 10.3390/metabo11120887

**Published:** 2021-12-18

**Authors:** Shohei Akagawa, Yuko Akagawa, Sohsaku Yamanouchi, Yoshiki Teramoto, Masahiro Yasuda, Sadayuki Fujishiro, Jiro Kino, Masato Hirabayashi, Kenji Mine, Takahisa Kimata, Masaki Hashiyada, Atsushi Akane, Shoji Tsuji, Kazunari Kaneko

**Affiliations:** 1 Department of Pediatrics, Kansai Medical University, Hirakata-shi, Osaka 573-1010, Japan; akagawas@hirakata.kmu.ac.jp (S.A.); akagaway@hirakata.kmu.ac.jp (Y.A.); yamanous@hirakata.kmu.ac.jp (S.Y.); yosh_teramoto@yahoo.co.jp (Y.T.); yasudams1@gmail.com (M.Y.); chupachappii@yahoo.co.jp (S.F.); kinojr.co.jp@gmail.com (J.K.); hirabaym@hirakata.kmu.ac.jp (M.H.); minek@hirakata.kmu.ac.jp (K.M.); kimatat@hirakata.kmu.ac.jp (T.K.); tsujis@hirakata.kmu.ac.jp (S.T.); 2 Department of Legal Medicine, Kansai Medical University, Hirakata-shi, Osaka 573-1010, Japan; hashiyam@hirakata.kmu.ac.jp (M.H.); akane@hirakata.kmu.ac.jp (A.A.)

**Keywords:** microbiota, neonatal jaundice, bilirubin, 16S rRNA gene sequencing, Bifidobacteriales

## Abstract

Neonatal jaundice, caused by excess serum bilirubin levels, is a common condition in neonates. Imbalance in the gut microbiota is believed to play a role in the development of neonatal jaundice. Thus, we aimed to reveal the gut microbiota characteristics in neonates with jaundice. 16S rRNA gene sequencing was performed on stool samples collected on day 4 from 26 neonates with jaundice (serum total bilirubin > 15.0 mg/dL) and 17 neonates without jaundice (total serum bilirubin < 10.0 mg/dL). All neonates were born full term, with normal weight, by vaginal delivery, and were breastfed. Neonates who were administered antibiotics, had serum direct bilirubin levels above 1 mg/dL, or had conditions possibly leading to hemolytic anemia were excluded. The median serum bilirubin was 16.0 mg/dL (interquartile range: 15.5–16.8) and 7.4 mg/dL (interquartile range: 6.8–8.3) for the jaundice and non-jaundice groups, respectively. There was no difference in the alpha diversity indices. Meanwhile, in the jaundice group, linear discriminant analysis effect size revealed that Bifidobacteriales were decreased at the order level, while Enterococcaceae were increased and Bifidobacteriaceae were decreased at the family level. Bifidobacteriaceae may act preventatively because of their suppressive effect on beta-glucuronidase, leading to accelerated deconjugation of conjugated bilirubin in the intestine. In summary, neonates with jaundice had dysbiosis characterized by a decreased abundance of Bifidobacteriales.

## 1. Introduction

Neonatal jaundice is a common condition that affects up to 80% of newborn babies [1,2]; it is caused by high levels of bilirubin in the sera, and most commonly appears within several days of birth. Severe neonatal jaundice has recently decreased, owing to advances in disease detection methods and treatment devices. However, bilirubin encephalopathy caused by hyperbilirubinemia is still reported globally, especially in developing countries [3,4].

The onset of neonatal jaundice is determined by multiple factors, including physiology, isoimmunization (Rh sensitization), genetic alteration, and environmental factors [5]. Recently, the imbalance of gut microbiota—known as dysbiosis—has also been considered as a pathogenic factor for neonatal jaundice [6,7,8,9]. In addition, it has been reported that a history of neonatal jaundice elevates the future risk of various diseases, including allergic diseases [10,11,12,13], type I diabetes [14], and autism spectrum disorder [15], although the mechanistic link has not been investigated. Interestingly, the development of these diseases is also related to dysbiosis [16,17,18,19,20]. Therefore, it can be hypothesized that the dysbiosis occurring immediately after birth may be the cause of various diseases, including neonatal jaundice, rather than neonatal jaundice itself increasing the risk of other diseases. Therefore, in this study, we determined whether neonates with jaundice have dysbiosis, and analyzed the characteristics of the gut microbiota in neonates with jaundice.

## 2. Results

### 2.1. Participants’ Characteristics

The NJ (neonatal jaundice) group included 26 neonates, of which 14 were male (54%), and the median gestational age was 272 days (interquartile range (IQR): 267–275). The HC (healthy control) group included 17 neonates, of which 10 were male (59%), and the median gestational age was 275 days (IQR: 269–281). There were no significant differences in sex or gestational age between the two groups. All neonates were born by vaginal delivery and were breastfed; however, in cases where the breast milk supply was insufficient, formula was administered. There were no significant differences in the Apgar score, maternal age, gravidity, parity, or maternal blood type between the groups. Furthermore, no mothers had premature rupture of the membrane or had taken antibiotics during the four weeks prior to delivery. The median serum bilirubin on day 4 was 16.0 mg/dL (IQR: 15.5–16.8) for the NJ group and 7.4 (IQR: 6.8–8.3) for the HC group, with a significant difference between the two groups (*p* < 0.001; Table 1).

### 2.2. Alpha Diversity

**Figure 1:**
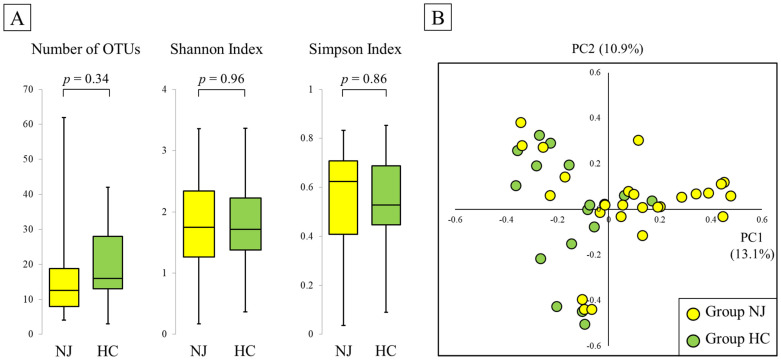
Alpha and beta diversity in neonatal jaundice (NJ) and healthy control (HC) groups: (**A**) Number of operational taxonomic units (OTUs), Shannon index, and Simpson’s index. The bottom and top edges of the boxes represent the 25th and 75th percentiles, respectively. Central vertical lines extend to the maximum and minimum values. (**B**) Principal coordinates analysis plot of Bray–Curtis dissimilarity. Each point represents a sample. Yellow points represent the NJ group, while green points represent the HC group. The visible and apparent clustering distances reveal the distinct structures of the gut microbiota in the two groups.

There was no significant difference in the number of operational taxonomic units (OTUs) between the NJ (13 (IQR, 8–19)) and HC (16 (13–28)) groups (*p* = 0.34). Moreover, there were no significant differences in the Shannon and Simpson indices (Shannon index: 1.75 (1.26–2.24) and 1.71 (1.38–2.22), *p* = 0.96; Simpson’s index: 0.62 (0.41–0.71) and 0.53 (0.45–0.69), *p* = 0.86, for NJ and HC, respectively; Figure 1A).

### 2.3. Beta Diversity

To evaluate the difference in gut microbiota composition between the NJ and HC groups, a principal coordinates analysis plot of Bray–Curtis dissimilarity was used for both groups to characterize the samples in two dimensions. The visible and apparent clustering distances revealed distinct gut microbiota structures in the NJ and HC groups (Figure 1B).

### 2.4. Taxonomic Composition

**Figure 2:**
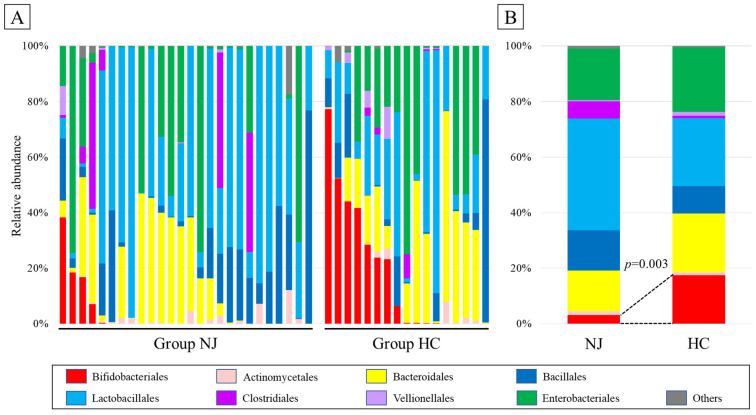
Composition of the gut microbiota of the neonatal jaundice (NJ) and healthy control (HC) groups at the order level. Each bar represents an individual (**A**) or a group (**B**). The proportion (relative abundance, in %) of Bifidobacteriales was significantly lower in the NJ group.

At the order level, Lactobacillales were the most dominant bacteria in both groups. Although the difference was not statistically significant, the trend indicated more Lactobacillales in the NJ group (*p* = 0.16). Furthermore, the percentage of Bifidobacteriales was significantly higher in the NJ group as compared with the HC group (17.5% vs. 3.1%, respectively, *p* = 0.003; Figure 2).

### 2.5. Linear Discriminant Analysis Effect Size (LEfSe)

**Figure 3:**
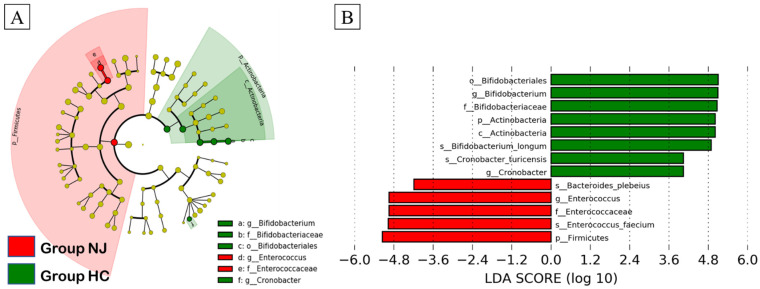
Linear discriminant analysis effect size (LEfSe) of gut microbiota in neonatal jaundice (NJ) and healthy control (HC) groups. (**A**) Cladogram generated by LEfSe indicating differences between the two groups at the phylum, class, order, family, and genus levels. Each successive circle represents a phylogenetic level. Regions in red indicate taxa enriched in the NJ group, while regions in green indicate taxa enriched in the HC group. Differing taxa are listed on the right-hand side of the cladogram. (**B**) Histogram of LDA scores calculated for selected taxa, showing significant differences in microbe type and abundance between the NJ (red) and HC (green) groups. LDA scores on the log 10 scale are indicated at the bottom.

A linear discriminant analysis (LDA) effect-size-based cladogram, in which successive circles represent phylogenetic levels (e.g., phylum, class, order, family, genus), showed that the taxa that were enriched in the NJ group belonged to the phylum Firmicutes, family Enterococcaceae, and genus *Enterococcus*. Conversely, taxa that were enriched in the HC group belonged to the phylum Actinobacteria, class Actinobacteria, order Bifidobacteriales, family Bifidobacteriaceae, and genera *Bifidobacterium* and *Cronobacter* (Figure 3A). In the LDA histogram that included species, the NJ group was enriched in *Enterococcus faecium* and *Bacteroides plebeius*, whereas *Bifidobacterium* *longum* and *Cronobacter turicensis* were significantly decreased (Figure 3B). 

### 2.6. Post Hoc Power Analysis

A post hoc power analysis was conducted to evaluate the outcome of the relative abundance of Bifidobacteriales. The post hoc power analysis yielded an effect size of 0.802 and a power of 0.795, with type I error of 0.05 and sample sizes of 26 and 17 for the NJ and HC groups, respectively. The power was almost equivalent to 0.8, which was set based on an a priori power analysis.

## 3. Discussion

In this study, we compared the gut microbiota of 26 neonatal patients with jaundice and 17 healthy neonates using 16S rRNA gene sequencing, and found that neonatal patients with jaundice had fewer bacteria belonging to the order Bifidobacteriales. Additionally, LEfSe identified a greater abundance of Enterococcaceae in neonatal patients with jaundice, although this result was not statistically significant.

To date, three studies have investigated the characteristics of the gut microbiota in children with neonatal jaundice using 16S rRNA sequencing. Duan et al. [6] analyzed stool samples collected between day 14 and day 35 after birth from 12 neonates with jaundice and 22 neonates without jaundice [6]; they reported that the total proportion of *Escherichia* and *Shigella* was significantly lower in neonates with jaundice. Li et al. [7] analyzed stool samples collected between day 0 and day 28 after birth from 10 neonates with jaundice and 10 neonates without jaundice, and reported that bacteria belonging to Proteobacteria were more abundant, while bacteria belonging to Firmicutes were less abundant, in neonates with jaundice [7]. Zhou et al. [8] compared 16 neonates with jaundice and 14 neonates without jaundice, and concluded that neonates with jaundice had fewer *Bifidobacterium* in their gut microbiota [8]. In the present study, there were no significant differences in alpha diversity between groups. Duan et al. [6] reported that only the Simpson’s index value was lower in neonates with jaundice [6]. However, another two studies reported no alpha diversity difference in neonates with jaundice [7,8]. As for beta diversity, in our study, neonates with jaundice presented different clusters from the healthy controls. Li et al. [7] found no difference in beta diversity [7], whereas Zhou et al. [8] observed differences, demonstrating that the literature to date reports mixed results with regard to beta diversity. As shown, no large-scale study has been conducted, and no common characteristics have been reported in the performed studies. One of the important issues causing a difference in the results may be the different inclusion/exclusion criteria and the difference in the timing of sample collection among studies. Since the gut microbiota of neonates changes drastically after birth, even one week of difference can lead to significant differences in both bacterial load and bacterial composition [21,22,23]. Therefore, it is crucial to unify the timing of sampling when analyzing the gut microbiota in neonates. In this study, we limited the timing of stool sampling to 96–120 h after birth (day 4). In addition, we used strict inclusion criteria, such as not including neonates whose mother’s blood type was O, so as to avoid the possible effect of mild hemolytic jaundice due to blood group incompatibility, or neonates with cephalohematoma, so as to eliminate the factors that cause hemolytic anemia.

Gut microbiota play a crucial role in enterohepatic circulation [24]. Unconjugated bilirubin, primarily derived from heme, is separated from albumin and enters the hepatocytes; it is then conjugated to glucuronide by UGT1A1 and excreted into the intestine, where it is deconjugated by beta-glucuronidase derived from certain anaerobic bacteria, such as *Escherichia coli*, *Peptostreptococcus*, *Bacteroides*, or *Clostridium* [25]. From there, it is either excreted through urine and feces or reabsorbed in the liver via the bloodstream. Increased heme due to high hemoglobin levels, insufficient activity of UGT1A1, and immature gut microbiota are considered to be the factors of potential hyperbilirubinemia in newborns. Importantly, higher activity of beta-glucuronidase leads to accelerated deconjugation of the conjugated bilirubin in the intestine and faster bilirubin absorption, subsequently elevating bilirubin levels in the blood. Notably, *Bifidobacterium* is reported to suppress beta-glucuronidase activity [26], and therefore acts preventatively on the development of jaundice. Therefore, a decreased abundance of Bifidobacteriales in the NJ group in our study may be a factor accelerating hyperbilirubinemia. On the other hand, the direct effect of bilirubin on bacteria was studied by Nobles et al. [27], who reported that bilirubin suppresses the growth of *Enterococcus faecalis* through membrane destabilization. The increased abundance of Enterococcaceae in children with jaundice might be explained by the decreased bilirubin levels in the intestine resulting from the accelerated absorption of bilirubin. Overall, dysbiosis characterized by decreased Bifidobacteriales and increased *Enterococcus* may be a factor affecting the onset of neonatal jaundice.

It has been previously reported that neonatal jaundice and phototherapy elevate the future risk of various diseases, including allergic diseases [10,11,12,13], type I diabetes [14], and autism spectrum disorder [15]. However, the mechanistic link between neonatal jaundice and phototherapy has not been investigated. This study and previous similar studies present the possibility of underlying dysbiosis in patients with neonatal jaundice. Considering the strong evidence that dysbiosis is associated with a wide variety of conditions, including the aforementioned conditions [16,17,18,19,20], it may be possible that neonatal jaundice and phototherapy do not directly relate to the onset of such conditions, but are confounding factors. To confirm the association between dysbiosis and jaundice, a longitudinal study of the microbiomes of infants with jaundice should be conducted.

Our study has several limitations. First, we did not assess the actual causal relationship between elevated Bifidobacteriales and neonatal jaundice. Functional genomic analysis using whole-genome sequencing, phylogenetic investigation of communities via the reconstruction of unobserved states analysis followed by quantitative polymerase chain reaction (PCR), or the measurement of beta-glucuronidase activity are recommended in order to clarify the nature of this relationship. Second, we were not able to reveal how differences in bacterial composition occurred between the groups. Although we aligned the major factors that affect the establishment of the gut microbiota in neonates, the effects of other factors—including genetic factors, the maternal microbiome, and the small amount of formula—were not analyzed. Furthermore, although all participants were breastfed on day 4, this does not mean that the neonates were exclusively breastfed, because some amount of formula may have been added when the midwife assessed that the breastmilk supply was insufficient. Nutrition in early life greatly affects the gut microbiota—particularly the presence of Bifidobacteriales [28]. The fact that participants were not exclusively breastfed may have biased our results. Third, we could not achieve the target sample size of 20 for the HC group, which was set based on a priori power analysis. However, the post hoc power analysis yielded a power of 0.795 when type I error was set at 0.05, with sample sizes of 26 and 17 for the NJ and HC groups, respectively. This suggests that although the sample size was small in the present study, it was sufficient for the analysis. However, a future study with a larger sample size is recommended. Lastly, we recruited neonates from only one region of Japan; hence, our findings may not be applicable to other areas or ethnic groups. However, because the incidence of jaundice varies greatly by race and nationality, our results are useful as a genuine microbiota profile for Japanese people.

In conclusion, neonates with jaundice were found to have dysbiosis characterized by a decreased abundance of Bifidobacteriales. Further studies are warranted to explore the potential of the gut microbiota as a therapeutic target for preventing various diseases, including neonatal jaundice.

## 4. Materials and Methods

### 4.1. Participants and Study Design

This study was conducted among children born between April 2020 and September 2020 at Kansai Medical University, Osaka, Japan. A total of 26 children whose measured total serum bilirubin at day 4 (96–120 h after birth) check-up was above 15 mg/dL were recruited as the NJ group, whereas 17 children with total serum bilirubin below 10 mg/dL were recruited as the HC control group. All mothers and their babies after vaginal delivery were discharged on day 5, even if they had no medical issues. All participants were recruited from the general baby ward, and were not treated for any clinical problems. Children born before 37 weeks, born after 42 weeks, born with a birth weight below 2500 g, born with a birth weight above 4000 g, born via cesarean section, those who had not been fed breastmilk on day 4, those who were administered antibiotics, those who had a premature rupture of the membrane, those who were admitted to the neonatal intensive care unit, those whose mother’s blood type was type O or Rh minus, those whose mother was positive for irregular antibodies, those who had direct bilirubin levels above 1 mg/dL, those who had cephalohematoma or subgaleal hemorrhage, those who showed predicted thyroid dysfunction or predicted hemolytic anemia, and/or those who had received phototherapy for jaundice before day 4 were not included.

Stool samples were collected on day 4 for 16S rRNA sequencing. Alpha and beta diversities and relative abundance of bacteria at the order level were compared between the two groups, and linear discriminant analysis effect size (LEfSe) was also performed.

The study was conducted in accordance with the guidelines of the Declaration of Helsinki, and approved by the Ethics Committee of Kansai Medical University (No. 2019257). Informed consent was obtained from all participants before enrollment.

### 4.2. Blood Sampling and Total Serum Bilirubin Measurements

Forty microliters of capillary whole-blood samples were routinely collected via a heel prick on day 4 for the measurement of total serum bilirubin. Total serum bilirubin was measured within 10 min of blood sampling by the optical density method using Bilmeter F (Atom Medical Corp., Tokyo, Japan). Blood sampling and measurements were conducted by an experienced neonatologist or gynecologist.

### 4.3. Stool Sampling and 16S rRNA Gene Sequencing

Stool samples (1 g) were collected 96–120 h after birth (day 4) from a disposable diaper using a sterilized spoon provided with the container, and stored at −80 °C within 2 h of sampling. The samples were thawed, and DNA was extracted using a NucleoSpin DNA Stool Kit (MACHEREY-NAGEL, Düren, Germany). Hypervariable regions of the DNA, and v2, v3, v4, v6-7, v8, and v9 of the 16S rRNA region, were multiplex amplified using a 16S metagenomics kit, following the manufacturer’s instructions (Thermo Fisher Scientific, Waltham, MA, USA). A library was generated after refinement using the Ion Plus Fragment Library Kit and Ion Xpress Barcode Adapters Kit (Thermo Fisher Scientific). The Ion Universal Library Quantification Kit with the Quant Studio 5 system (Thermo Fisher Scientific) was used to quantify and pool the barcoded library in order to generate a final concentration of 50 pM per target. The Ion Chef Instrument and the corresponding kit were used to achieve target concentrations for template preparation and emulsion PCR. Sequence analysis was performed using the Ion Gene Studio S5 System and Ion 530 chip (Thermo Fisher Scientific). The sequences were then clustered into OTUs with 97% identity using the MicroSEQ 16S Reference Library v2013.1 and Greengenes v13.5 databases. The raw BAM file was analyzed using Ion Reporter Software with the Metagenomics 16S w1.1 v5.16 workflow (Thermo Fisher Scientific), with default settings (read length filter: 150; minimum alignment coverage: 90.0; read abundance filter: 10; slush ID reporting percentage: 0.2). Ion Reporter leverages Quantitative Insights Into Microbial Ecology (QIIME)’s open-source bioinformatics pipeline, which was used to produce alpha and beta diversity analyses and visualizations.

### 4.4. LEfSe

The LEfSe algorithm was used to identify the taxa that differed in relative abundance between the two groups [29]. The Online Galaxy version 1.0 interface (http://huttenhower.sph.harvard.edu; accessed on 28 August 2021) was used. The alpha value for the factorial Kruskal–Wallis test among classes was set to 0.05, and the threshold on the logarithmic LDA score for discriminative features was set to 3.5.

### 4.5. Statistical Analysis and Sample Size

Continuous variables are represented as the median and IQR, while categorical variables are represented as numbers and percentages. Chi-squared and Mann–Whitney U tests were used for comparisons between the two study groups. Statistical significance was set at *p* < 0.05. To calculate the appropriate sample size for the study, we conducted a priori power analysis for the relative abundance of Bifidobacteriales using G*Power version 3.1.9.4 (Heinrich Heine University, Düsseldorf, Germany) [30], with an effect size of 0.8 and a type I error of 0.05. Since a sample size of 20 for each group would provide a power of 0.8, we set the target participants as 20 for each group. To verify the results, a post hoc power analysis for the relative abundance of Bifidobacteriales was performed, with a type I error of 0.05. All other statistical analyses were performed with BellCurve for Excel (version 3.21; Social Survey Research Information Co., Ltd., Tokyo, Japan).

## Figures and Tables

**Table 1:** General information regarding the participants with and without jaundice.

Characteristics	Group NJ (*n* = 26)	Group HC (*n* = 17)	*p*-Value
Sex, male (%)	14 (54%)	10 (59%)	0.75
Gestational age (days)	272 (267–275)	275 (269–281)	0.22
Birth weight (g)	3118 (2773–3385)	3255 (3094–3410)	0.16
Delivery mode, vaginal delivery (%)	26 (100%)	17 (100%)	1.00
Nutrition, breastfed (%)	26 (100%)	17 (100%)	1.00
Use of antibiotics after birth (%)	0 (0%)	0 (%)	1.00
Apgar score 1 min	8 (8–9)	8 (8–9)	0.51
Apgar score 5 min	9 (9–9)	9 (9–9)	0.06
Maternal age (years)	34 (29–38)	32 (30–35)	0.67
Gravidity	1 (1–2)	2 (1–2)	0.11
Parity	0 (0–1)	1 (0–1)	0.07
Premature rupture of the membrane	0 (0%)	0 (%)	1.00
Use of antibiotics by mothers during the four weeks prior to delivery	0 (0%)	0 (%)	1.00
Maternal blood type			
A	17 (65%)	11 (65%)	0.96
B	6 (23%)	5 (29%)	0.70
AB	3 (12%)	1 (6%)	0.53
O	0 (0%)	0 (0%)	1.00
Day 4 serum bilirubin (mg/dL)	16.0 (15.5–16.8)	7.4 (6.8–8.3)	<0.001

Data are expressed as the number (%) or median (interquartile range). NJ: neonatal jaundice; HC: healthy controls.

## Data Availability

The datasets used and analyzed during this study can be found in the Kansai Medical University Research Data Storage, and are available from the corresponding author upon reasonable request. The data are not publicly available because of privacy restrictions.
